# Revealing the novel complexity of plant long non-coding RNA by strand-specific and whole transcriptome sequencing for evolutionarily representative plant species

**DOI:** 10.1186/s12864-022-08602-9

**Published:** 2022-05-19

**Authors:** Yan Zhu, Longxian Chen, Xiangna Hong, Han Shi, Xuan Li

**Affiliations:** 1 Key Laboratory of Synthetic Biology, Center for Excellence in Molecular Plant Sciences/Institute of Plant Physiology and Ecology, Chinese Academy of Sciences, Shanghai, China; 2 University of Chinese Academy of Sciences, Beijing, China; 3 Henan University, Kaifeng, China

**Keywords:** lncRNA, lincRNA, lncNAT, Plant, Strand-specific

## Abstract

**Background:**

Previous studies on plant long noncoding RNAs (lncRNAs) lacked consistency and suffered from many factors like heterogeneous data sources and experimental protocols, different plant tissues, inconsistent bioinformatics pipelines, etc. For example, the sequencing of RNAs with poly(A) tails excluded a large portion of lncRNAs without poly(A), and use of regular RNA-sequencing technique did not distinguish transcripts’ direction for lncRNAs. The current study was designed to systematically discover and analyze lncRNAs across eight evolutionarily representative plant species, using strand-specific (directional) and whole transcriptome sequencing (RiboMinus) technique.

**Results:**

A total of 39,945 lncRNAs (25,350 lincRNAs and 14,595 lncNATs) were identified, which showed molecular features of lncRNAs that are consistent across divergent plant species but different from those of mRNA. Further, transposable elements (TEs) were found to play key roles in the origination of lncRNA, as significantly large number of lncRNAs were found to contain TEs in gene body and promoter region, and transcription of many lncRNAs was driven by TE promoters. The lncRNA sequences were divergent even in closely related species, and most plant lncRNAs were genus/species-specific, amid rapid turnover in evolution. Evaluated with PhastCons scores, plant lncRNAs showed similar conservation level to that of intergenic sequences, suggesting that most lincRNAs were young and with short evolutionary age. INDUCED BY PHOSPHATE STARVATION (*IPS*) was found so far to be the only plant lncRNA group with conserved motifs, which may play important roles in the adaptation of terrestrial life during migration from aquatic to terrestrial. Most highly and specially expressed lncRNAs formed co-expression network with coding genes, and their functions were believed to be closely related to their co-expression genes.

**Conclusion:**

The study revealed novel features and complexity of lncRNAs in plants through systematic analysis, providing important insights into the origination and evolution of plant lncRNAs.

**Supplementary Information:**

The online version contains supplementary material available at 10.1186/s12864-022-08602-9.

## Background

Long noncoding RNAs (lncRNAs) were found to account for a large part of the whole transcriptome in plants. Most of lncRNAs identified in plants were lincRNAs, which were transcribed from intergenic regions. Another lncRNAs transcribed from intragenic regions were lncNATs, which were antisense to coding genes. Both types of lncRNAs have been identified in many plant species, among which a few were found to play important roles in plant development and stress resistance [1–4]. Benefiting from the high-throughput sequencing technology, bulks of RNA-sequencing data were deposited to public databases. Databases of plant lncRNAs have been developed in the past several years, such as PLncDB, CANTATAdb, GreeNC, NONCODE, et al. [5–8]. More than one million lncRNAs were recorded in these databases, which covered more than 80 plant species. However, a systematic analysis on plant lncRNA evolution has not been conducted due to many difficulties. The heterogeneous data sources and protocols, like inconsistent plant tissues, ways of sequencing library construction, pipelines for bioinformatics analysis, were cited as some of the factors [9–12]. Most of the previous studies sequenced RNAs with poly(A) tails by oligo(dT) selection, which would exclude a large portion of lncRNAs without poly(A) [13, 14]. In addition, the regular RNA-sequencing technique does not distinguish transcripts’ direction, which would lack a large part of lncRNAs antisense to coding genes. Therefore, to comprehensively analyze the lncRNAs in divergent plant species and their evolution requires 1) unified study design with consistent plant sources, and 2) the whole transcriptome RNA-sequencing technology with strand-specificity.

To date, important question about how plants lncRNAs originated and evolved remains largely un-answered. Several ways for the origin of lncRNAs have been documented, among which transposable elements (TEs) are an important cause. TE was thought as an important factor to drive the transcription of lncRNAs [15, 16]. In humans, about 75% lncRNAs contained at least one exon deriving from TEs [15]. In plants, several lncRNAs were found to originate from TEs [16, 17]. *lncRNA-314* was originated from a full-length LTR retrotransposon inserting into downstream of a promoter in the ancestral tomato genome [16]. In *Arabidopsis*, *lincRNA11195* contained a LTR retrotransposon and was activated under abiotic stresses [17]. In addition to these two plant lncRNAs, more research is needed to understand the roles of TEs in the origination of lncRNAs in plant.

LncRNAs had a fast evolutionary rate and a high degree of sequence diversity in animals [18–20]. Some ancient lncRNAs were highly conserved in tetrapod, and they were preserved in evolution and may have important functions [20]. Plant lncRNAs were highly divergent at the nucleotide level [21]. LncRNAs showed highly divergency on sequences between rice and maize, and between *Brassicaceae* and *Cleomaceae* [22, 23]. Besides, molecular features of plant lncRNAs did not follow the classic evolutionary patterns, and they showed little evidence of phylogenetic relationships [24]. A comprehensive design is needed to systematically study the plant lncRNA evolution and feature divergency.

The current study was designed to comprehensively discover and analyze lncRNAs in multiple plant species across the evolutionary landscape using strand-specific and whole transcriptome sequencing data. We employed eight plant species from the primitive to higher taxa in plant, which were representatives at key position of plant evolution. Using RiboMinus and strand-specific RNA-sequencing techniques in combination with consistent plant tissues, we have expanded the lncRNA landscape, including long noncoding natural antisense RNAs (lncNATs) and lncRNAs without poly(A). Our results showed that both lincRNAs and lncNATs were widely distributed in plants. TEs played important roles in the origination and transcription of lncRNAs. The molecular features of lncRNAs were found to be conserved in plants, but sequences were non-conserved even between evolutionarily close species. Most highly and specially expressing lncRNAs participated in the co-expressional network with coding genes, which may function as regulators in the network.

## Results

### Identification of plant lncRNAs with new lncNAT species from strand-specific RiboMinus transcriptome sequencing data

**Fig. 1:**
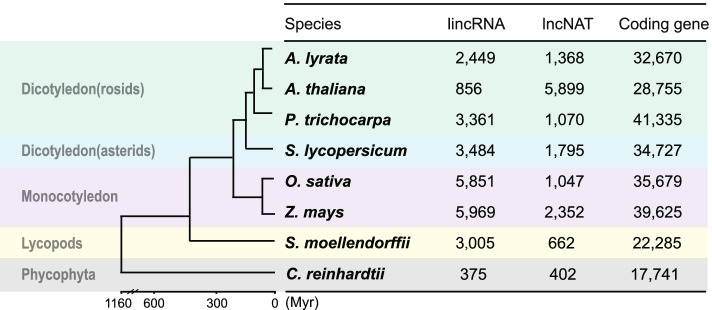
Phylogenetic relationship of plant species in this study and lncRNAs identified in each species. The phylogenetic tree was drawn using TimeTree (http://www.timetree.org/). Myr: million years

To systematically study the molecular features and evolutionary profiles of lncRNAs in plants, we selected eight representative plant species across the phylogenetic landscape for analysis, including *Chlamydomonas reinhardtii* (*C. reinhardtii*), *Selaginella moellendorffii* (*S. moellendorffii*), *Zea mays* (maize), *Oryza sativa* subsp*. Japonica* (rice), *Arabidopsis lyrata* (*A. lyrata*), *Arabidopsis thaliana* (*A. thaliana*), *Populus trichocarpa* (poplar) and *Solanum lycopersicum* (tomato) (Fig. 1). These species can represent single-cell alga, ferns, monocotyledon and dicotyledon, spanned an evolutionary history of 116 million years. Whole-genome sequences and annotations of these species were available from public databases (Additional file 1: Table 1).

Different from previous studies, we acquired only RiboMinus and strand-specific RNA-seq (ssRNA-seq) data for multiple plant tissues, either collected from public database or sequenced by ourselves when they are not available (Additional file 2). This critical approach was designed to include lncRNA species either with poly(A) or without poly(A), which can also identify the transcriptional orientation of lncRNAs, and their location on sense- or antisense- strand of coding gene regions. A consensus and efficient lncRNA identification workflow were constructed based on our previous study [25]. To reduce the variation emerging from the tissue difference among species, we selected data of five tissues: root, stem, leaf, flower, and seed/fruit. For lower-level plant species without differentiated tissues, such as *C. reinhardtii* and *S. moellendorffii*, we used all available data. Considering the batch effects coming from PCR artifacts, sequence depth and gene expressional abundance, etc. among different data sources, we eliminated PCR artifacts and low-quality sequencing data by pre-processing and mapping high-quality reads to genome in our procedure. We required the mapped data size for each tissue to be above 10X genomic coverage depth in order to retain transcripts with low-level expression (“Additional file 2” listed the data size of each sample for all tissues of the eight species). We normalized the expression of transcripts using FPKM, which reduced or eliminated the discrepancy from sequencing depth and transcript length. At last, we removed those low-expressional transcripts (FPKM 0.5 for single-exon transcripts and 0.1 for multiple-exon transcripts) to resolve the transcripts with low expressional abundance. For species with annotations including lncRNAs, we added them in our result if those lncRNAs satisfied our criterion.

A total of 39,945 lncRNAs were obtained in all the eight species, for which the transcriptional orientation of each lncRNA was also confirmed. LncRNAs identified in this study were categorized into two types: lncRNA in intergenic region (lincRNA: 25,350) and lncRNA antisense to one or more exons of coding genes (lncNAT: 14,595) (Fig. 1, Additional file 1: Table 2, Additional file 3). Both of lincRNAs and lncNATs dispersed widely in all of the plant clades from 777, the least number in *C. reinhardtii* to 8321, the most in maize. By comparing lncRNAs identified in this study with lncRNAs from the databases of PLncDB, CANTATAdb, GreeNC, and NONCODE, a total of 22,313 new lncRNAs (12,838 lincRNAs and 9475 lncNATs) were found, and their distribution in each species were listed in “Additional file 1: Table 2”.

Previous studies on plant lncRNAs often missed out lncNATs due to lack of orientation in their RNA sequencing data (thus could not made accurate distinction on lncNATs from coding gene transcripts). Here we searched lncNATs using strand specific RNA-seq data, and found that lncNATs dispersed widely in plant (Fig. 1). LncNATs took a large part in plant lncRNAs, and even more than lincRNA in several species, such as *A. thaliana* and *C. reinhardtii* (Fig. 1). There are about 20% coding genes with lncNATs transcribing in *A. thaliana*. Coding genes can be expressed with their counterpart lncNATs simultaneously (Additional file 4: Fig. S1A). Compared with coding genes without lncNATs, their expressional levels were not significantly affected by the presence of lncNATs (Additional file 4: Fig. S1B). The lack of correlation between the expression of coding genes and their paired lncNATs implied the lncNATs may not be directly involved in the regulation of coding genes’ expression.

### The consistent molecular features of lncRNAs among divergent plant species

The characteristics of lncRNAs, such as transcript length, AT content, exon number, and so on, were explored and compared to coding genes in individual plant species [11, 26, 27]. However, if these features were consistent in plants were not clear. In addition to the most important differences between lncRNA and mRNA that was coding products, other features remained to be investigated between lncRNA and mRNA in plants.

**Fig. 2:**
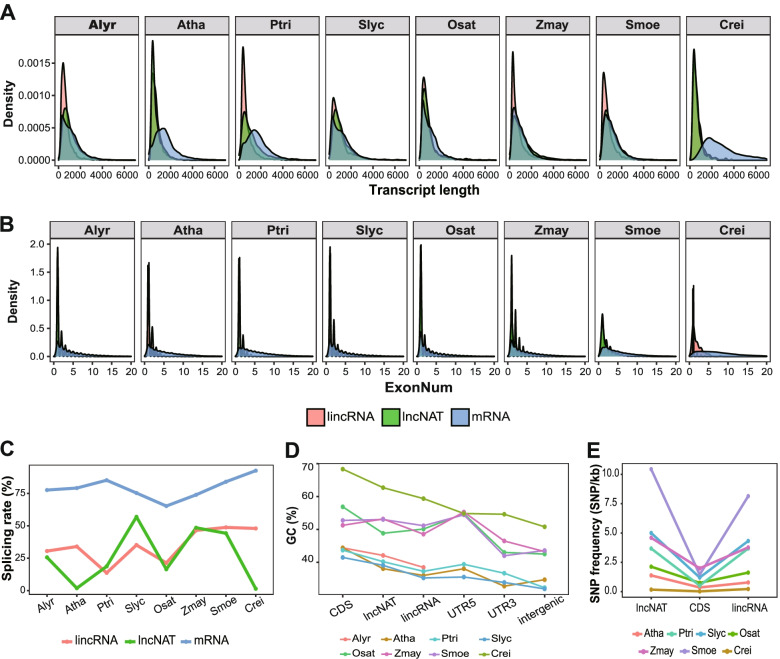
Novel conserved features of lncRNAs in plant. **A**, **B** Length distribution and Exon-number of lincRNA, lncNAT and mRNA in each species. **C** Splicing ratio of lincRNA, lncNAT and mRNA in each species. **D** GC content of lincRNA, lncNAT, CDS/5’UTR/3’UTR of coding genes and intergenic sequences in each species. **E** SNP frequency of lincRNA, lncNAT and CDS of coding genes in each species

In current study, we revealed comprehensive characteristics of lncRNAs in multiple species using unified sequencing data and bioinformatic analysis procedure. We found both lncNATs and lincRNAs have more simple structures than mRNAs. LncRNAs were shorter than mRNAs (median length of lncNAT: 964, lincRNA: 817, mRNA: 1422, both *p*-value < 2.2e-16, t-test) (Fig. 2A), and lncRNAs have fewer exons compared to mRNAs (Mean of lncNAT: 1.4, lincRNA: 1.7, mRNA: 5.2, both *p*-value < 2.2e-16, t-test) (Fig. 2B). These two features were conserved in plant lncRNAs (Fig. 2A, B). LncRNAs existed in plant genomes mainly as single-exon transcripts. A total of 65% lincRNAs and 82% lncNATs were single-exon, while in mRNAs this number was 22%. This phenomenon was observed in all plant species of this study, and was in consistent with results of other plant species in previous studies [27].

Fewer multiple-exon transcripts meant few requirements for splicing by lncRNA. In a previous study, researchers found lncRNAs were rarely spliced and mainly non-polyadenylated [28]. The splicing efficiency in human and mouse showed that inefficient splicing might be a common feature of lncRNAs across species [29]. The ratio of splicing lncRNAs was significantly lower than those for mRNAs (Wilcoxon test, both *p*-value = 7.6e-06, Additional file 4: Fig. S2A) in this study. Splicing lincRNAs ranged from 13.7% (poplar) to 48.8% (*S. moellendorffii*), compared to the ratio of mRNAs from 65.3% (rice) to 92.6% (*C. reinhardtii*) (Fig. 2C). Inefficient splicing of lncRNAs was not just a feature in animals but is also common across plant species. Next, we wanted to investigate if the splicing sites in lncRNAs were same with mRNAs. Further analysis of the base distribution upstream and downstream of splicing sites showed that sequences of splice sites in lncRNAs were conserved across plant species. They have almost the same sequence context between lncRNAs and mRNAs, which suggested that they probably used the same splicing mechanism (Additional file 4: Fig. S2B).

Both of lincRNAs and lncNATs have conserved nucleotide constitution among multiple species. LncRNAs have lower GC content than coding sequences (CDS) of coding genes, while which was higher than intergenic sequences (Fig. 2D). This feature was also consistent across multiple species in plant. High GC content was usually associated with coding sequences [30]. Though lncNATs and coding genes shared overlapping sequences, the GC content of lncNATs was still lower than CDS in most plant species (Fig. 2D). The bias of nucleotide in lncRNA meant the selective pressure on their sequences. At the same time, mutants accumulated in both lincRNAs and lncNATs were much higher than coding genes (Fig. 2E, Additional file 1: Table 3). Mutants accumulated in coding regions with the possibility to change amino acid and lead to the change of protein products. While the absence of coding products for lncRNAs made them tolerate more mutants. Although lncNATs shared sequences with coding genes, their sequences accumulated more mutants and fewer GC bases, implied they may experience different paths to coding genes in evolution.

### New discovery of lncRNA origination by inserting of transposable elements (TEs)

**Fig. 3:**
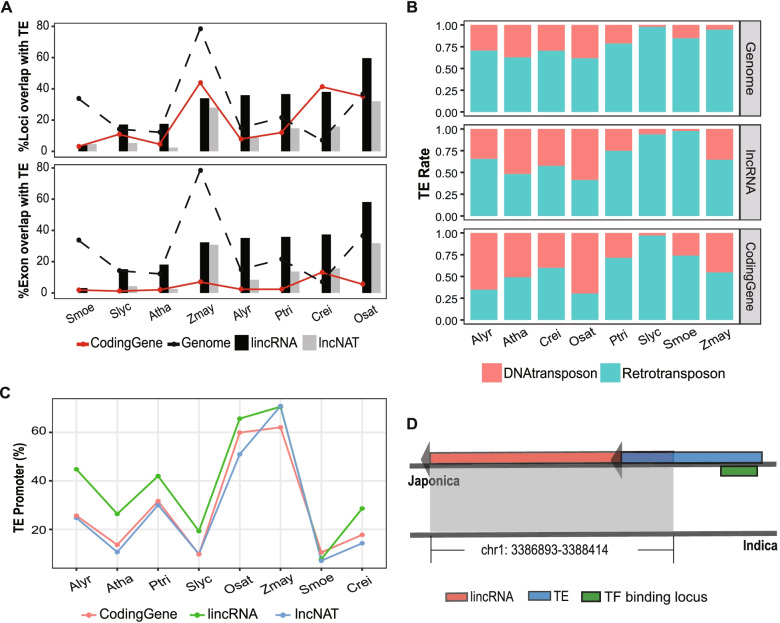
TEs regulate the transcription of lncRNAs. **A** Genomic loci of lncRNAs and coding genes overlapping with TEs (up). Exonic sequences of lncRNAs and coding genes overlapping with TEs (down). The black dashed lines represent the ratio of TE sequences in whole genome; the red solid lines represent the ratio of coding genes overlapping with TEs; and the black and grey bars represent the ratio of lincRNA and lncNAT overlapping with TEs, respectively. **B** The distribution of Class I and Class II TEs in genome, lncRNAs and coding genes. **C** The ratio of lncRNAs with TE promoters in each species. **D** TE-lincRNA *Osat_00007032* in rice (subspecies: Japonica). The up black line represents genome of Japonica, while the down one represents genome of Indica. The grey block between Japonica and Indica was the syntenic area between the two genomes. The locus of *Osat_00007032* was signed with the red and blue bar, while the syntenic area in Indica was not found with lncRNAs or other elements. The TE upstream *Osat_0000732* belonged to MERMITEJ sub-family, and was a type of DNA/Gypsy TE

TEs were widely existed in eukaryotic genomes and acted as key factors for gene and genome evolution [31, 32]. Insertion of TEs was considered as one of many ways for origin of lncRNA [15, 33]. To study the roles of TE for lncRNAs, we first established a reference library of TEs for each genome using RepeatMasker [34]. As a result, up to 59.7% lincRNAs can be found contain TEs in their sequences (Fig. 3A). Besides, we found a large proportion of TEs inserted into the genomic loci of lncRNAs and coding genes (lincRNA: 3.9% ~ 59.7%, lncNAT: 2.4% ~ 32%, coding gene: 3.1% ~ 43.9%). But, when we limited the inserting regions to exons of lncRNAs and CDS of coding genes, the ratio of transcripts overlapping with TEs decreased sharply in the latter (1.8% ~ 13.1%), while in lncRNAs the ratio remained (lincRNA: 3.1% ~ 58.2%, lncNAT: 0.5% ~ 31.8%) (Fig. 3A). Significantly, sequences of TEs were remained in lncNATs though they shared sequences with coding genes. Decrease of TEs in protein coding sequences indicated that TEs inserting into CDS were eliminated in evolution since they may change gene products, and resulted in serious consequences. In coding genes, TE insertion was concentrated in regions with limited effects on gene products, such as UTR, intron. On the contrary, lncRNAs were not influenced by insertion of TEs in their exons for lack of coding products, so these TE sequences can be kept and accompanied by lncRNAs in evolution. This suggested that lncRNAs may originate from TE insertion. The close relationship between lncRNA and TEs revealed the key roles of TEs played in the origin of lncRNA.

TEs were classified into two types: Class I TEs or retrotransposons, and Class II TEs or DNA transposons. They are both present in the plant genome. However, we found that Class II TEs favored transcribed regions, including lncNAT and lincRNA, whereas Class I TEs favored regions of non-transcribed sequences (Fig. 3B). The ratio between Classes I and II TEs deviating from that the entire genome indicated that the TEs, Classes I and II, in lncRNAs and coding sequences were under different selection pressure.

TEs play important roles on the origination of lncRNAs. Benefitting from the strand-specific sequencing data, we can confirm the positions of promoters. We found about 38 ± 21% lincRNAs had promoters originating from TEs, which was higher than the percentage of coding genes (29 ± 20%) and lncNATs (27 ± 21%) (Fig. 3C). The ratio of lncRNAs with promoters originating from TEs was positively related to whole genomic TE content in each species (Pearson’s correlation, lincRNA: 0.63, lncNAT: 0.83). This suggested that TEs randomly inserted into genome was one of the major ways to generate lncRNAs.

Further, we found TEs initiated the transcription of lncRNAs by supplying TF binding sequences for lncRNAs (Fig. 3D). Combining with ChIP-seq data from nine transcript factors (TFs) in *A. thaliana*, *A. lyrata*, rice and maize, we identified 250 lncRNAs, which contained TF binding sites originating from TEs, and these lncRNAs accounted for 11.7% of lncRNAs with TF biding sites being confirmed (Additional file 1: Table 4, Additional file 5). As an example, we found a lincRNA *Osat_00007032*, which was upstream of coding gene *OS01G0166800* in rice (subsp. Japonica), and expressed in anther, pistil, leaf, root, stem and seed, but especially high in pistil (Additional file 4: Fig. S3). A TE belonging to MERMITEJ subfamily covered the upstream of *Osat_00007032*, and provided promoter for *Osat_00007032*. In the promoter, binding sequences for transcript factor MADS29 was confirmed (Fig. 3D). MADS29 belongs to MADS-box transcription factors, which were critical regulators for rice reproductive development [35, 36]. Surprisingly, we cannot detect lncRNAs or TEs in the syntenic region of genome for Indica, which was another subspecies of rice. This indicated that lincRNA *Osat_00007032* was specific for Japonica, and was evolved recently by TE insertion. This novel discovery that TE brought promoters containing TF binding sites to the upstream exonic locus, revealed the relationship between TE and lncRNA different from that previously found in tomato lncRNA, for which a TE inserted into the downstream of a promoter [16].

### Most plant lincRNAs only conserved within genus indicating rapid turnover in evolution

**Fig. 4:**
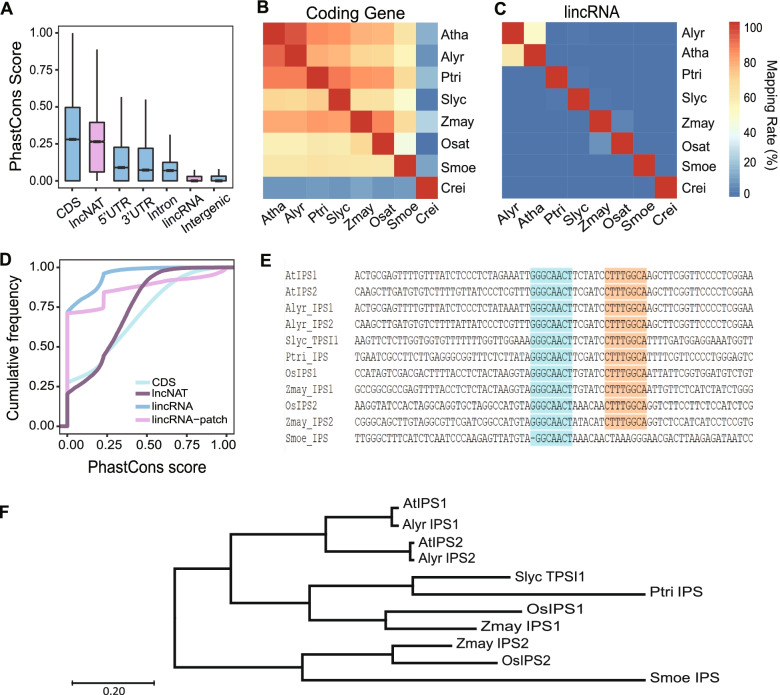
The conservation of lncRNAs in plant. **A** The PhastCons scores of coding genes (including CDS, 5’UTR, 3’UTR, and Intron), lncRNAs (lincRNA and lncNAT) and intergenic sequences. **B**, **C** Conserved lincRNAs and coding genes in each species. LincRNAs were aligned to genome of another species using blastn, while amino acid sequences of coding genes were aligned to another species’ amino acid sequences using blastp. The numbers of legend were the ratio of transcripts with homologous sequences in the genome of other species. The labels were abbreviations of each species. **D** The cumulative frequency of PhastCons scores in coding genes and lncRNAs. The lincRNA-patch meant a window (12 bp) sliding from the first base to the last one, and then the highest window score was selected to stand for the score of the lincRNA. **E** Highly conserved *IPS* motifs in all plant species in this study except algae. The marked sequences with orange and turquoise were regions combining to miRNA. **F** The phylogenetic relationships of terrestrial plant was constructed according to the nucleotide sequences of *IPS* using MEGA7 (https://www.megasoftware.net/)

Sequence conservation provides insight into evolutionary process of functional elements in genome. However, most lncRNAs in plants were found with poor conservation, including the lncRNAs whose functions were well studied [37, 38]. In this study, we attempted to explore the evolution of lncRNAs in plants by analyzing the conservation of lncRNAs with two methods. First, we evaluated the sequence conservation using PhastCons score [39]. PhastCons score was calculated based on the result of seven-way whole genomic alignment for *A. thaliana*, *A. lyrata*, soybean, rice, poplar, tomato and *S. moellendorffii*. We compared the sequence conservation of lincRNAs, lncNATs, CDS of coding genes, 5’UTR and 3’UTR of coding genes, and used sequences from intergenic regions as control. As expected, CDS exhibited the highest sequence conservation, while the intergenic sequence was the lowest (Fig. 4A). Considering that lncNATs and coding genes shared some common sequences, they showed similar sequence conservation (Fig. 4A, Additional file 4: Fig. S4A). The conservation of lincRNAs, however, was lower than 5’UTR, 3’UTR and introns of coding genes (Fig. 4A). The proportion of plant lincRNAs with PhastCons scores higher than 0.6 was only 0.2%, and this number in coding genes was 14.4%. About 71% plant lincRNAs scored 0, indicating very low conservation (Additional file 4: Fig. S4B). In placental mammals, old (minimum age 90 Myr) and young lncRNAs (minimum age 25 Myr) were defined basing on the phylogenetic distribution of species, and they found the conservation of old lncRNAs were close to CDS, while young lncRNAs were even lower than intergenic regions [20]. In this study, we found plant lincRNAs showed similar conservation to intergenic sequences, and which suggested that most plant lincRNAs were young and with short evolutionary age (Fig. 4A, Additional file 4: Fig. S4A).

Further, we evaluated the conservation of plant lincRNAs by identifying the homologous lncRNAs across the eight plant species. We mapped lincRNAs in one species to genomes of all other species. LncNATs were excluded since the existence of overlapped regions shared with coding genes. While strong conversation of coding genes existed between evolutionarily remote species in the plant kingdom, the conservation of lincRNAs retained only in the evolutionarily close groups (Fig. 4B, C). Previous studies of lncRNAs showed that the lncRNA homology mainly existed within a family in both plants and animals [20, 21]. However, we found conserved lincRNAs were rare in plant family, as only 5% lincRNAs from rice were homologous with those in maize (Fig. 4C). In genus such as *Arabidopsis*, half of lincRNAs were found having homologs in other species within the same genus (Fig. 4C). Hence, we found that most of the homologous lincRNAs were genus- or species-specific in plants, suggesting that lincRNAs have a rapid turnover in evolution.

### *IPS* was found to be the only plant lncRNA group with conserved sequence motifs

Some lincRNAs were found to be functional via short-conserved patches, though the whole sequences were divergent [40, 41]. To know if conserved patches existed in plant lncRNAs, we developed a method using sliding windows to calculate the mean PhastCons score for each patch. We found most lincRNAs have no apparently conserved patches in the eight plant species (Fig. 4D). We then looked into the plant lncRNAs in lncrnadb [42], and found *IPS* was the only lncRNA with conserved patches.

*IPS* lncRNA family participated in phosphorus (P_i_) homeostasis in plants, including *AtIPS1* and *AtIPS2*/*At4* in *Arabidopsis*, *OsIPS1* and *OsIPS2* in rice, and *TPSI1* in tomato [43–45]. They were found to be accumulated under Pi starved condition. *IPS* contained conserved 24-nt nucleotides motif in several species [43, 45]. The motif contained two highly conserved regions which were separated by three nucleotides, and played roles as the target mimicry for miR399 [46]. Strikingly, our study found all species contained this motif except algae (Fig. 4E). The absence of *IPS* in algae may be explained by their lack of vascular tissues, whereas *IPS* was expressed in vascular tissues when lacking of P_i_. *IPS* first appeared in *S. moellendorffii*, a representative of primitive vascular plant (Fig. 4E, F). But only one of the two *IPS* motifs was found in *S. moellendorffii*, whereas two motifs were often found in high plants (Fig. 4E, F). Different from most lncRNAs with short evolutionary histories, *IPS* underwent a long evolutionary history with conserved function in P_i_ homeostasis, suggesting plant lncRNAs may play important roles in the adaptation of terrestrial life during migration from aquatic to terrestrial.

### Specific expression of lncRNAs revealing sophisticated transcription regulation

**Fig. 5:**
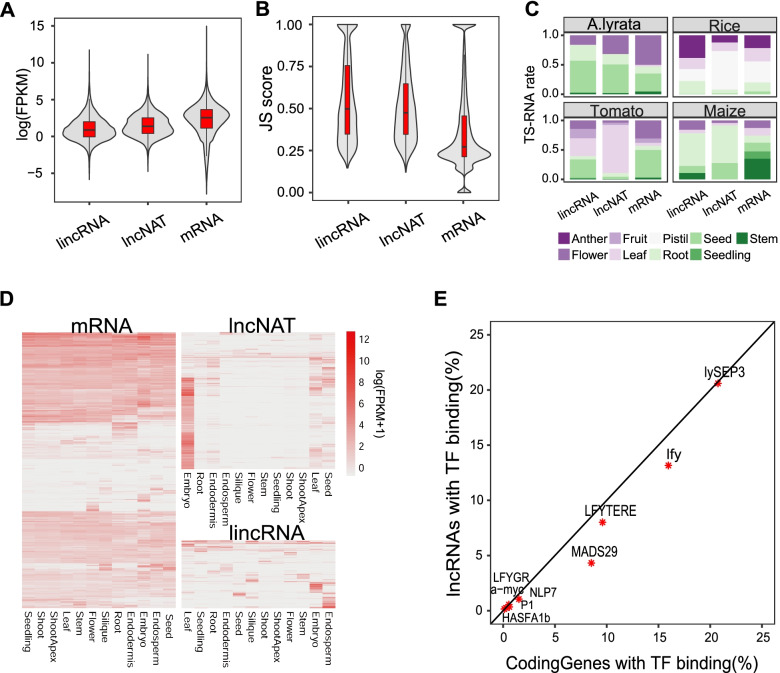
The expressional patterns of lncRNAs in plant. **A**, **B** Expressional levels (FPKM) and tissue specificity (JS score) of lincRNA, lncNAT and mRNA. **C** Tissue distribution of TS (tissue-specific) transcripts in several plant species **D** Tissue distribution of all expressed transcripts in *A. thaliana*. The legend numbers were calculated using log(FPKM+ 1). **E** Transcript factor binding frequency of lncRNA and mRNA

Previously several lncRNA studies showed that most lncRNAs were expressed at low levels, and often expressed in specific tissues or conditions in plants [11, 26]. In this study, we attempted to profile the expression of lincRNAs and lncNATs in more species. Both lincRNA and lncNAT showed lower expression levels and higher tissue specificity compared to coding genes (Fig. 5A, B). LincRNA and lncNAT showed similar expressional levels (average: 35.2 vs 22.9), while the expressional level of mRNA was significantly higher (average: 124.8, Kolmogorov-Smirnov test, both *p*-value < 2.2e-16) (Fig. 5A). Similar trends were observed in all plant species (Additional file 4: Fig. S5A). Low expressional level was a common feature for plant lncRNAs.

Tissue specificity was evaluated using Jensen-Shannon (JS) score in all of the plant species, which returned a score between 0 and 1 [47]. JS score of 1 represented the transcript expressed uniquely in one tissue, while JS score of 0 meant the transcript expressed in all tissues. Both lincRNA and lncNAT exhibited significantly higher JS score, i.e., higher tissue specificity, than mRNA (Kolmogorov-Smirnov test, both *p*-value < 2.2e-16) (Fig. 5B, Additional file 4: Fig. S5B).

The expressional patterns of lncRNAs evolved rapidly in plant, which formed sharp contrast to the coding genes. We defined tissue-specific (TS) transcripts as transcript with JS score > = 0.9. We found TS lncRNAs and TS mRNAs were distributed to tissues differently in the same species (Fig. 5C). Also, among the different plants, TS lncRNAs of tomato and maize were more likely to express in leaf and root, while TS lncRNAs were found mostly in the pistil of rice, and the seed of *A. lyrata*.

Previous studies in rice and maize showed that lncRNAs were more specially expressed in reproductive tissues [11, 26]. By comparing the expression of all transcripts, we found mRNAs were more evenly expressed in all tissues in *A. thaliana*, while lncRNAs were significantly different among tissues (Fig. 5D). In *A. thaliana*, lncNATs were preferentially expressed in embryo, while lincRNAs were in both endosperm and embryo. LncRNAs had a narrow expression profile compared to coding genes.

LncRNAs showed similar TF binding rate compared to that of coding genes, though they had low expressional levels and unstable expression patterns. We used ChIP-seq data of nine transcript factors (TFs) from *A. thaliana*, *A. lyrata*, rice and maize to predict the binding sites in promoter (Additional file 5). The frequency of lncRNA promoters binding with TFs was comparable to coding genes (Fig. 5E). TFs with low binding rate in coding genes also had low binding efficiency in lncRNA, e.g., *LFYGR*, *NLP7*, *P1*, *a-myc*, *HASF1b*.

### Plant lncRNAs forming co-expression network with coding genes

**Fig. 6:**
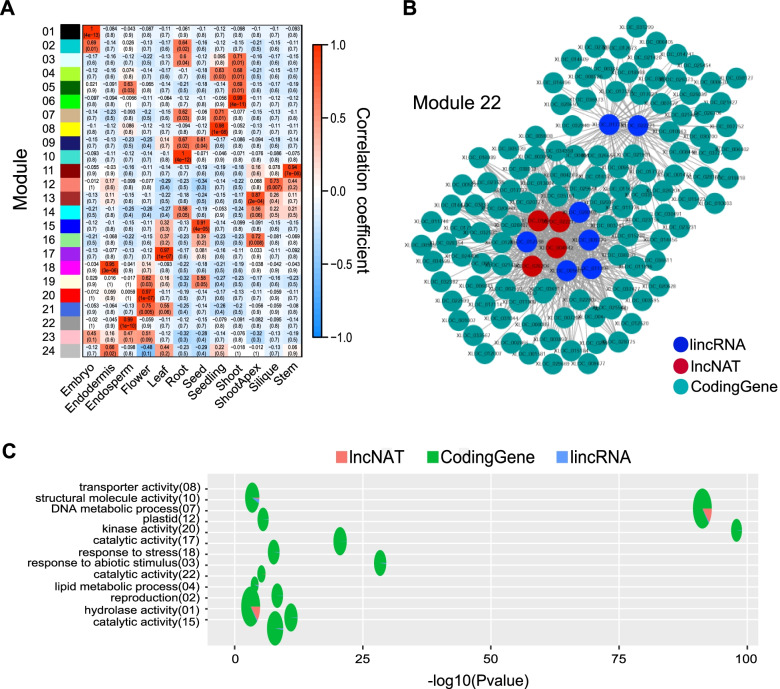
The WGCNA network of lncRNAs and coding genes, and function prediction of lncRNAs co-expressed with coding genes. **A** Correlation matrix between modules and tissues of *A. thaliana*. **B** Correlational network of the module 22. The red and blue circles represent lncNAT and lincRNA respectively, while the other circles represent coding genes. **C** Functional description of modules containing lncRNAs. The area of the pie was positively correlated to the number of transcripts. The numbers in the brackets correspond to the modules in (**A**)

Expression is often closely related to function, especially highly and specifically expressed lncRNAs [48, 49]. We selected lncRNAs that were highly expressed in one or two tissues in *A. thaliana* to construct WGCNA network. A total of 178 lincRNAs, 555 lncNATs and 20,729 coding genes were selected to construct the network. In this network, lncRNAs and coding genes were clustered into 24 modules according to their expressional characteristics (Fig. 6A). In these modules, a total of 689 lncRNAs (155 lincRNAs and 534 lncNATs) were found to be co-expressed with coding genes. Especially, lncNATs in the network were not directly related to their antisense coding genes, which referred they may not regulate the transcription of antisense genes. GO enrichment analysis was performed for the coding genes in each module. Functions of the coding genes were summarized as follows: I) Basic metabolism: kinase activity, lipid metabolic process, and hydrolase activity; II) Response to stress and response to abiotic stimulus; III) Reproduction (Fig. 6C). LncRNAs involved in the network may play roles in these functions.

The correlation between modules and samples showed that the expressional patterns of lncRNAs were highly correlated to specific tissues (Fig. 6A). In our result, lncRNAs of *A. thaliana* were highly and specially expressed in embryo and endosperm (Fig. 5D). In WGCNA network, the embryo was strongly correlated with module 01, and the endosperm was strongly correlated to module 22 (Fig. 6A, B). In module 01, we found a myriad of coding genes associating with embryo development. Functions of these genes can be categorized as: I) Involving in embryo development directly; II) Overexpression, mutation or knocking down caused embryo death; III) Enrichment of protein products during embryonic development. Co-expressional relationship was found between these coding genes and lncRNA, and lncRNAs in this network may play roles in embryo development. In module 22, two radial networks were constituted of four lncNATs and seven lincRNAs with coding genes (Fig. 6B). In these coding genes, we found four genes encoding transcript factors played roles in the endosperm development. As an example, gene *AT1G02580* encoded transcript factor *MEDEA/MEA*, a polycomb group gene that was imprinted in the endosperm [50]. All of the 11 lncRNAs in the network were found co-expressed with it, which indicating that these lncRNAs may play roles in endosperm imprint. Coincidently, researchers found some lncRNAs expressing in castor bean seeds were involved in genomic imprint, and several of them comprised the imprinted cluster with imprinted coding genes [27].

## Discussion

Benefiting from the advancement of high-throughput sequencing technology, we have discovered more lncRNAs in representative plant species that are associated with novel complexity. Most of the previous studies sequenced RNAs with poly(A) tails by oligo(dT) selection, which would exclude a large part of lncRNAs without poly(A) [14]. In addition, lncNATs was much more difficult to study with the non-strand specific RNA-sequencing technology, whose distribution in plant was largely not clear. Here we studied lncRNAs using RiboMinus and strand-specific RNA-sequencing data. We expanded the plant lncRNA landscape, and found the lncNATs dispersed widely in plant. The numbers of lncNATs were even greater than lincRNAs in several species, such as *A. thaliana* and *C. reinhardtii*. Although several lncNATs were reported to regulate the expression of antisense coding genes [51, 52], we found most lncNATs were not correlated to the expression of antisense coding genes (Additional file 4: Fig. S1B). This observation implied the function of most lncNATs may not be related to their complimentary coding genes.

In plants, insertion of TEs is one of the important mechanisms for lncRNA origin [16, 53]. We found many plant lncRNAs from all the studied species contained sequence elements of TEs. In *A. thaliana*, rice and maize, the proportion of TE-related lincRNAs in total lincRNAs were reported to range from 22.9 to 51.5% [17], similar to our results (18.2% ~ 58.2%) on these species. TEs were mainly inserted into the exons of plant lncRNAs, avoiding exons with coding genes (Fig. 3A). Particularly, we found an example that TE insertion caused initiating new transcription, which has not been reported in plants. This finding provided further evidence that TEs play key roles in the origin of plant lncRNAs. Note several different ways for the origin of lncRNAs were documented, including coding genes losing coding capacity, genomic sequences devoid of exons and insertion of TEs [54]. Our research added new details for lncRNAs origination with novel complexity.

Some lncRNAs were found to be conserved in animals with the species were distantly separated in evolution [20, 55]. These ‘old’ lncRNAs that were preserved in animals, were suggested to have important functions [20]. However, plant lncRNAs were highly divergent, and almost all plant lncRNAs were species/genus-specific. Differently from animals, we did not find any plant lncRNAs that are conserved along the evolution. Our results agree with others’ studies that did not detect highly conserved plant lncRNAs [21, 22].

Conserved sequence patches within lncRNAs were previously reported in some lncRNAs [40]. *IPS* was a lncRNA participating in Pi regulation in several plant species, and was induced expressing in the vascular tissues of root and shoot [41, 45]. However, we have found the highly conserved motifs of *IPS* crossed all tracheophyte (Fig. 4E, F). The emergency of *IPS* in all terrestrial plants indicated that *IPS* lncRNAs may play important roles in the adaptative evolution of plant migrating from aquatic environments to terrestrial ones.

To date, the functions of a large number of lncRNAs in plants remain unknown. Our finding that most highly and specially expressed lncRNAs formed co-expression network with coding genes suggested they may play roles in the plant development, stress response, etc. For example, they may participate in embryo development and endosperm imprint. However, within the large amount of lncRNAs identified in plant species, only a small portion of them were found in potential functional networks. The large numbers of novel plant lncRNAs found in our study open an avenue to systematically characterize and explore the functions and evolution of lncRNAs in plant.

## Conclusions

We performed RiboMinus combining with strand-specific RNA-sequencing technique on eight evolutionarily representative plant species. We identified 39,945 lncRNAs, which including 14,595 lncNATs, and expounded the distribution of lncNATs in plants. Further, we found TEs played key roles in the origination of plant lncRNAs. Combing with ChIP-seq data, we found a new way for lncRNA originating from TE insertion. In addition, we found plant lncRNAs were not conserved in evolutionarily distant species, and most of them were species/genus-specific. However, conserved motifs of *IPS* were found in lncRNA of all terrestrial plants, and *IPS* may played important roles in the adaptive evolution of plant. The study revealed novel features and complexity of lncRNAs in plants through systematic analysis, providing important insights into the origination and evolution of plant lncRNAs.

## Methods

### Plant materials

The seeds of *A. thaliana* and *A. lyrata* were buy from ABRC (https://abrc.osu.edu/users/sign_in?redirect_to=%2Forders%2Fnew), and then cultivated in phytotron respectively. Leaves, stems, roots and flowers of *A. thaliana* and *A. lyrata* were collected from mature plants. Siliques of *A. thaliana* were collected one week after flowering, and seeds were collected when siliques were full but epidermis were still green. The seedlings of poplar were provided by Cuiting Wang, CAS Center for Excellence in Molecular Plant Sciences. Leaves, stems, roots and shoot tips of poplar were collected from seedlings with height of ~ 1 m. The mature plants of rice (Japonica) were obtained from the lab of Zuhua He, CAS Center for Excellence in Molecular Plant Sciences (http://sippe.ac.cn/zuhuahe/). Leaves and stems of rice were collected before heading period. Samples of *S. moellendorffii* were described in our previous study [25].

### RNA sequencing and data acquisition

Our dataset contained more than 700G read pairs from eight plant species, of which 490G were previously published data and 210G were generated by RNA sequencing (Additional file 2). Data downloaded from public databases can be found by the accession numbers recording in “Additional file 2”. Samples been sequenced in this study were dealt with following processes: materials were taken from at least two plant individuals, and then been mixed for RNA isolation. Total RNA was extracted, and rRNA was removed from the purified RNA using Ribo-Zero rRNA Removal Kit. Strand-specific RNA-seq libraries were constructed with TruSeq Stranded mRNA LT Sample Prep Kit. Libraries were applied to Illumina Hiseq2500 platform.

### Bioinformatics pipeline for identification of lncRNAs

Sequencing reads were filtered according to the quality score using sickle (version 1.210), and the quality threshold was set as 20. Reads shorter than 50 bp or with ambiguous nucleotides were removed after quality checking. Genomes and gene sets of the eight plant species were downloaded from public databases, and the versions of them were shown in “Additional file 1: Table 1”. High quality reads were aligned to the genomes of each species using TopHat2 (version 2.1.0) [56]. Cufflinks (version 2.2.1) was used to construct transcripts of each sample according to the mapping results [57]. The expressional levels of transcripts were evaluated by FPKM using Cufflinks software suite. JS score was employed to represent the tissue specificity, and which was calculated using R library cummeRbund (version 2.7.2). We defined a transcript as lncRNA if it satisfied the following criteria: I) it must be longer than 200 bp; II) it was noncoding by the result of CPC and PLEK [58, 59]; III) it had no homologs in databases of Swiss-Prot [60], Pfam [61] or Rfam [62]; IV) the FPKM of it was more than 0.5 for single-exon transcript or more than 0.1 for multiple-exon transcript.

### Characterization of lncRNAs and coding genes

#### SNP calling

All clean reads were mapped to genome using bwa (version 0.7.17-r1188) [63]. Samtools (version 1.9) and bcftools (version 1.9) were used to call SNPs [64]. All SNPs from various tissues or libraries were merged, then SNPs with quality more than 10 and depth more than 5 were retained. At last, high-quality SNPs in the region of lncRNAs and coding genes were retained using local scripts.

#### Transcript factor binding site identification

ChIP-seq data from *A. thaliana*, *A. lyrata*, rice and maize were downloaded from SRA database, and their accession numbers were listed in “Additional file 5”. After quality filtering using sickle (version 1.210), clean reads were mapped to genome using bowtie2 (version 2.2.6) [65], and the parameter was set as -N 1. Peak calling was subjected to MACS (version 2.1.2) with module “callpeak” (version 2.1.2), and the parameters were set as --keep-dup 1 and -q 0.01 [66].

#### TE annotation

Whole genomic repeats and TEs of the eight plant species in this study were identified using RepeatMasker 3.3.0 with parameters setting as -xsmall, and the RepBase20.02 were used as reference library. TE-lncRNAs were defined as lncRNAs with exon sequence overlapping at least 5 bp to TE sequences.

### PhastCons score

Lastz (version 1.02.00) was used for inter-species alignments, then TBA (version v12) was used for final integration to obtain genome-wide alignment files for all eight species [67]. PhastCons score (PCs) were calculated using phast (version 1.3) according to the results of genome-wide alignment [39]. Conserved patches in lncRNAs were defined as short patches (12 bp) with PCs more than 0.6, and meanwhile PCs of the whole lncRNAs were less than 0.3.

### WGCNA network construction

The WGCNA co-expressional network was constructed with R package WGCNA (version 1.68) using the FPKMs from all the 12 tissues of selected genes in *A. thaliana* [68]. Genes complying with the following criteria were selected to construct the network: 1) lncNATs expressing in one or two tissues, and their expression levels were among the top 10%; 2) lincRNAs expressing in one or two tissues, and with which the maximum FPKM was larger than 1; 3) mRNAs with FPKM larger than 1. The parameters of the key function “blockwiseModules” were set as: power = 16, mergeCutHeight = 0.25, and reassignThreshold = 0. The network of each module was showed by Cytoscape (Version 3.6.1) [69]. GO enrichment analysis was subjected by BiNGO (version 3.0.3), and the parameters were set as: the multiple testing correlation - “FDR correlation”, ontology file - “GOSlim_Plants”, and the organism annotation - “*Arabidopsis thaliana*” [70].

## Supplementary Information

**Additional file 1.**

**Additional file 2.** RNA-seq datasets.

**Additional file 3.** lncRNA-gff3.

**Additional file 4: Figure S1.** (A) Expressional levels of lncNATs and their antisense coding genes. The expressional levels were evaluated by log (FPKM+1). (B) Expressional levels of lncNAT, antisense coding genes, and coding genes without lncNATs expressing at their antisense strand. **Figure S2.** (A) Ratio of splicing and alternative splicing transcripts. (B) Base distribution of splicing acceptor and donor for lncRNAs and mRNAs. **Figure S3.** Expressional levels of lncRNA *Osat_00007032* in rice. **Figure S4**. The distribution of PhastCons score in lncRNAs and coding genes. (A) The distribution of PhastCons score in lncRNAs, intergenic sequences and coding sequences of coding genes. (B) Percentage of lncRNAs and mRNAs with different PhastCons scores in total lncRNAs and mRNAs, respectively. **Figure S5.** Expressional patterns of lncRNAs and mRNAs in divergent species. (A) Expressional levels of lncRNAs and mRNAs in each species, and the numbers of y-axes were calculated by log_10_(FPKM). (B) Tissue-specificity of lncRNAs and mRNAs in each species with multiple tissues, and the y-axes stands for JS score.

**Additional file 5.** ChIP-seq datasets.

## Data Availability

The raw sequence data reported in this paper have been deposited in the Genome Sequence Archive [71] in National Genomics Data Center [72], Beijing Institute of Genomics (China National Center for Bioinformation), Chinese Academy of Sciences, under accession number CRA003591 that are publicly accessible at https://bigd.big.ac.cn/gsa. The other RNA-seq raw data used in this study were downloaded from NCBI public database, and the accession numbers were listed in ‘Additional file 2’.

## Abbreviations

lncRNA: long noncoding RNA
lincRNA: long intergenic noncoding RNA
lncNAT: long noncoding natural antisense RNA
NAT: Natural antisense transcript
ncRNA: noncoding RNA
AS: Alternative splicing
TE: Transposable element
CDS: Coding sequence
TF: Transcript factor
PCs: PhastCons score
Pi: Phosphorus
FPKM: Fragment per kilobase per million mapped fragments
JS score: Jensen-Shannon score
TS: Tissue-special
WGCNA: Weighted correlation network analysis
GO: Gene ontology
Atha: *A. thaliana*
Alyr: *A. lyrata*
Ptri: *P. trichocarpa*
Slyc: *S. lycopersicum*
Zmay: *Z. mays*
Osat: *O. sativa*
Smoe: *S. moellendorffii*
Crei: *C. reinhardtii*

## References

[1] Liu X, Hao L, Li D, Zhu L, Hu S (2015). Long non-coding RNAs and their biological roles in plants. Genomics Proteomics Bioinformatics.

[2] Seo JS, Sun HX, Park BS, Huang CH, Yeh SD, Jung C, Chua NH (2017). ELF18-INDUCED LONG-NONCODING RNA associates with mediator to enhance expression of innate immune response genes in Arabidopsis. Plant Cell.

[3] Zhao X, Li J, Lian B, Gu H, Li Y, Qi Y (2018). Global identification of Arabidopsis lncRNAs reveals the regulation of MAF4 by a natural antisense RNA. Nat Commun.

[4] Sun Y, Hao P, Lv X, Tian J, Wang Y, Zhang X, Xu X, Han Z, Wu T (2020). A long non-coding apple RNA, MSTRG.85814.11, acts as a transcriptional enhancer of SAUR32 and contributes to the Fe-deficiency response. Plant J.

[5] Paytuvi Gallart A, Hermoso Pulido A, Anzar Martinez de Lagran I, Sanseverino W, Aiese Cigliano R (2016). GREENC: a Wiki-based database of plant lncRNAs. Nucleic Acids Res.

[6] Fang S, Zhang L, Guo J, Niu Y, Wu Y, Li H, Zhao L, Li X, Teng X, Sun X (2018). NONCODEV5: a comprehensive annotation database for long non-coding RNAs. Nucleic Acids Res.

[7] Jin J, Lu P, Xu Y, Li Z, Yu S, Liu J, Wang H, Chua NH, Cao P (2021). PLncDB V2.0: a comprehensive encyclopedia of plant long noncoding RNAs. Nucleic Acids Res.

[8] Szczesniak MW, Rosikiewicz W, Makalowska I (2016). CANTATAdb: A Collection of Plant Long Non-Coding RNAs. Plant Cell Physiol.

[9] Di C, Yuan J, Wu Y, Li J, Lin H, Hu L, Zhang T, Qi Y, Gerstein MB, Guo Y (2014). Characterization of stress-responsive lncRNAs in Arabidopsis thaliana by integrating expression, epigenetic and structural features. Plant J.

[10] Shumayla S, Taneja M, Tyagi S, Singh K, Upadhyay SK (2017). Survey of High Throughput RNA-Seq Data Reveals Potential Roles for lncRNAs during Development and Stress Response in Bread Wheat. Front Plant Sci.

[11] Li L, Eichten SR, Shimizu R, Petsch K, Yeh CT, Wu W, Chettoor AM, Givan SA, Cole RA, Fowler JE (2014). Genome-wide discovery and characterization of maize long non-coding RNAs. Genome Biol.

[12] Golicz AA, Singh MB, Bhalla PL (2018). The Long Intergenic Noncoding RNA (LincRNA) Landscape of the Soybean Genome. Plant Physiol.

[13] Djebali S, Davis CA, Merkel A, Dobin A, Lassmann T, Mortazavi A, Tanzer A, Lagarde J, Lin W, Schlesinger F (2012). Landscape of transcription in human cells. Nature.

[14] Yang L, Duff MO, Graveley BR, Carmichael GG, Chen LL (2011). Genomewide characterization of non-polyadenylated RNAs. Genome Biol.

[15] Kapusta A, Kronenberg Z, Lynch VJ, Zhuo X, Ramsay L, Bourque G, Yandell M, Feschotte C (2013). Transposable elements are major contributors to the origin, diversification, and regulation of vertebrate long noncoding RNAs. PLoS Genet.

[16] Wang X, Ai G, Zhang C, Cui L, Wang J, Li H, Zhang J, Ye Z (2016). Expression and diversification analysis reveals transposable elements play important roles in the origin of Lycopersicon-specific lncRNAs in tomato. New Phytol.

[17] Wang D, Qu Z, Yang L, Zhang Q, Liu ZH, Do T, Adelson DL, Wang ZY, Searle I, Zhu JK (2017). Transposable elements (TEs) contribute to stress-related long intergenic noncoding RNAs in plants. Plant J.

[18] Hezroni H, Koppstein D, Schwartz MG, Avrutin A, Bartel DP, Ulitsky I (2015). Principles of long noncoding RNA evolution derived from direct comparison of transcriptomes in 17 species. Cell Rep.

[19] Lopez-Ezquerra A, Harrison MC, Bornberg-Bauer E (2017). Comparative analysis of lincRNA in insect species. BMC Evol Biol.

[20] Necsulea A, Soumillon M, Warnefors M, Liechti A, Daish T, Zeller U, Baker JC, Grutzner F, Kaessmann H (2014). The evolution of lncRNA repertoires and expression patterns in tetrapods. Nature.

[21] Deng P, Liu S, Nie X, Weining S, Wu L (2018). Conservation analysis of long non-coding RNAs in plants. Sci China Life Sci.

[22] Mohammadin S, Edger PP, Pires JC, Schranz ME (2015). Positionally-conserved but sequence-diverged: identification of long non-coding RNAs in the Brassicaceae and Cleomaceae. BMC Plant Biol.

[23] Wang H, Niu QW, Wu HW, Liu J, Ye J, Yu N, Chua NH (2015). Analysis of non-coding transcriptome in rice and maize uncovers roles of conserved lncRNAs associated with agriculture traits. Plant J.

[24] Simopoulos CMA, Weretilnyk EA, Golding GB (2019). Molecular Traits of Long Non-protein Coding RNAs from Diverse Plant Species Show Little Evidence of Phylogenetic Relationships. G3 (Bethesda).

[25] Zhu Y, Chen L, Zhang C, Hao P, Jing X, Li X (2017). Global transcriptome analysis reveals extensive gene remodeling, alternative splicing and differential transcription profiles in non-seed vascular plant Selaginella moellendorffii. BMC Genomics.

[26] Zhang YC, Liao JY, Li ZY, Yu Y, Zhang JP, Li QF, Qu LH, Shu WS, Chen YQ (2014). Genome-wide screening and functional analysis identify a large number of long noncoding RNAs involved in the sexual reproduction of rice. Genome Biol.

[27] Xu W, Yang T, Wang B, Han B, Zhou H, Wang Y, Li DZ, Liu A (2018). Differential expression networks and inheritance patterns of long non-coding RNAs in castor bean seeds. Plant J.

[28] Schlackow M, Nojima T, Gomes T, Dhir A, Carmo-Fonseca M, Proudfoot NJ (2017). Distinctive Patterns of Transcription and RNA Processing for Human lincRNAs. Mol Cell.

[29] Mele M, Mattioli K, Mallard W, Shechner DM, Gerhardinger C, Rinn JL (2017). Chromatin environment, transcriptional regulation, and splicing distinguish lincRNAs and mRNAs. Genome Res.

[30] Pozzoli U, Menozzi G, Fumagalli M, Cereda M, Comi GP, Cagliani R, Bresolin N, Sironi M (2008). Both selective and neutral processes drive GC content evolution in the human genome. BMC Evol Biol.

[31] Bennetzen JL, Wang H (2014). The contributions of transposable elements to the structure, function, and evolution of plant genomes. Annu Rev Plant Biol.

[32] Slotkin RK, Martienssen R (2007). Transposable elements and the epigenetic regulation of the genome. Nat Rev Genet.

[33] Cho J (2018). Transposon-Derived Non-coding RNAs and Their Function in Plants. Front Plant Sci.

[34] Smit AF, Hubley R, Green P. Green: RepeatMasker Open-4.0. 2013-2015 <http://www.repeatmasker.org>.

[35] Yin LL, Xue HW (2012). The MADS29 transcription factor regulates the degradation of the nucellus and the nucellar projection during rice seed development. Plant Cell.

[36] Nayar S, Sharma R, Tyagi AK, Kapoor S (2013). Functional delineation of rice MADS29 reveals its role in embryo and endosperm development by affecting hormone homeostasis. J Exp Bot.

[37] Heo JB, Sung S (2011). Vernalization-mediated epigenetic silencing by a long intronic noncoding RNA. Science.

[38] Ding J, Lu Q, Ouyang Y, Mao H, Zhang P, Yao J, Xu C, Li X, Xiao J, Zhang Q (2012). A long noncoding RNA regulates photoperiod-sensitive male sterility, an essential component of hybrid rice. Proc Natl Acad Sci U S A.

[39] Siepel A, Bejerano G, Pedersen JS, Hinrichs AS, Hou M, Rosenbloom K, Clawson H, Spieth J, Hillier LW, Richards S (2005). Evolutionarily conserved elements in vertebrate, insect, worm, and yeast genomes. Genome Res.

[40] Lin N, Chang KY, Li Z, Gates K, Rana ZA, Dang J, Zhang D, Han T, Yang CS, Cunningham TJ (2014). An evolutionarily conserved long noncoding RNA TUNA controls pluripotency and neural lineage commitment. Mol Cell.

[41] Huang CY, Shirley N, Genc Y, Shi B, Langridge P (2011). Phosphate utilization efficiency correlates with expression of low-affinity phosphate transporters and noncoding RNA, IPS1, in barley. Plant Physiol.

[42] Quek XC, Thomson DW, Maag JLV, Bartonicek N, Signal B, Clark MB, Gloss BS, Dinger ME (2015). lncRNAdb v2.0: expanding the reference database for functional long noncoding RNAs. Nuncleic Acids Res.

[43] Hou XL, Wu P, Jiao FC, Jia QJ, Chen HM, Yu J, Song XW, Yi KK (2005). Regulation of the expression of *OsIPS1* and *OsIPS2* in rice via systemic and local Pi signalling and hormones. Plant Cell Environ.

[44] Liu C, Muchhal US (1997). Differential expression of TPS11, a phosphate starvation-induced gene in tomato. Plant Mol Biol.

[45] Shin H, Shin HS, Chen R, Harrison MJ (2006). Loss of At4 function impacts phosphate distribution between the roots and the shoots during phosphate starvation. Plant J.

[46] Franco-Zorrilla JM, Valli A, Todesco M, Mateos I, Puga MI, Rubio-Somoza I, Leyva A, Weigel D, Garcia JA, Paz-Ares J (2007). Target mimicry provides a new mechanism for regulation of microRNA activity. Nat Genet.

[47] Cabili MN, Trapnell C, Goff L, Koziol M, Tazon-Vega B, Regev A, Rinn JL (2011). Integrative annotation of human large intergenic noncoding RNAs reveals global properties and specific subclasses. Genes Dev.

[48] Cui J, Luan Y, Jiang N, Bao H, Meng J (2017). Comparative transcriptome analysis between resistant and susceptible tomato allows the identification of lncRNA16397 conferring resistance to Phytophthora infestans by co-expressing glutaredoxin. Plant J.

[49] Zhang G, Duan A, Zhang J, He C (2017). Genome-wide analysis of long non-coding RNAs at the mature stage of sea buckthorn (Hippophae rhamnoides Linn) fruit. Gene.

[50] Song Q, Ando A, Jiang N, Ikeda Y, Chen ZJ (2020). Single-cell RNA-seq analysis reveals ploidy-dependent and cell-specific transcriptome changes in Arabidopsis female gametophytes. Genome Biol.

[51] Yuan JH, Liu XN, Wang TT, Pan W, Tao QF, Zhou WP, Wang F, Sun SH (2017). The MBNL3 splicing factor promotes hepatocellular carcinoma by increasing PXN expression through the alternative splicing of lncRNA-PXN-AS1. Nat Cell Biol.

[52] Yue H, Zhu J, Xie S, Li F, Xu Q (2016). MDC1-AS, an antisense long noncoding RNA, regulates cell proliferation of glioma. Biomed Pharmacother.

[53] Cho J, Paszkowski J (2017). Regulation of rice root development by a retrotransposon acting as a microRNA sponge. Elife.

[54] Ponting CP, Oliver PL, Reik W (2009). Evolution and functions of long noncoding RNAs. Cell.

[55] Washietl S, Kellis M, Garber M (2014). Evolutionary dynamics and tissue specificity of human long noncoding RNAs in six mammals. Genome Res.

[56] Kim D, Pertea G, Trapnell C, Pimentel H, Kelley R, Salzberg SL (2013). TopHat2: accurate alignment of transcriptomes in the presence of insertions, deletions and gene fusions. Genome Biol.

[57] Trapnell C, Williams BA, Pertea G, Mortazavi A, Kwan G, van Baren MJ, Salzberg SL, Wold BJ, Pachter L (2010). Transcript assembly and quantification by RNA-Seq reveals unannotated transcripts and isoform switching during cell differentiation. Nat Biotechnol.

[58] Kong L, Zhang Y, Ye ZQ, Liu XQ, Zhao SQ, Wei L, Gao G (2007). CPC: assess the protein-coding potential of transcripts using sequence features and support vector machine. Nucleic Acids Res.

[59] Li A, Zhang J, Zhou Z (2014). PLEK a tool for predicting long non-coding RNAs and messenger RNAs based on an improved k-mer scheme. BMC Bioinformatics.

[60] UniProt C (2019). UniProt: a worldwide hub of protein knowledge. Nucleic Acids Res.

[61] El-Gebali S, Mistry J, Bateman A, Eddy SR, Luciani A, Potter SC, Qureshi M, Richardson LJ, Salazar GA, Smart A (2019). The Pfam protein families database in 2019. Nucleic Acids Res.

[62] Kalvari I, Argasinska J, Quinones-Olvera N, Nawrocki EP, Rivas E, Eddy SR, Bateman A, Finn RD, Petrov AI (2018). Rfam 13.0: shifting to a genome-centric resource for non-coding RNA families. Nucleic Acids Res.

[63] Li H, Durbin R (2009). Fast and accurate short read alignment with Burrows-Wheeler transform. Bioinformatics.

[64] Li H (2011). A statistical framework for SNP calling, mutation discovery, association mapping and population genetical parameter estimation from sequencing data. Bioinformatics.

[65] Langmead B, Salzberg SL (2012). Fast gapped-read alignment with Bowtie 2. Nat Methods.

[66] Feng J, Liu T, Qin B, Zhang Y, Liu XS (2012). Identifying ChIP-seq enrichment using MACS. Nat Protoc.

[67] Blanchette M, Kent WJ, Riemer C, Elnitski L, Smit AF, Roskin KM, Baertsch R, Rosenbloom K, Clawson H, Green ED (2004). Aligning multiple genomic sequences with the threaded blockset aligner. Genome Res.

[68] Langfelder P, Horvath S (2008). WGCNA: an R package for weighted correlation network analysis. BMC Bioinformatics.

[69] Shannon P, Markiel A, Ozier O, Baliga NS, Wang JT, Ramage D, Amin N, Schwikowski B, Ideker T (2003). Cytoscape: a software environment for integrated models of biomolecular interaction networks. Genome Res.

[70] Maere S, Heymans K, Kuiper M (2005). BiNGO: a Cytoscape plugin to assess overrepresentation of gene ontology categories in biological networks. Bioinformatics.

[71] Wang Y, Song F, Zhu J, Zhang S, Yang Y, Chen T, Tang B, Dong L, Ding N, Zhang Q (2017). GSA: Genome Sequence Archive. Genomics Proteomics Bioinformatics.

[72] National Genomics Data Center Members and Partners (2020). Database Resources of the National Genomics Data Center in 2020. Nucleic Acids Res.

