# Supplementary material for: Revealing the novel complexity of plant long non-coding RNA by strand-specific and whole transcriptome sequencing for evolutionarily representative plant species
Source: BMC Genomics. 2022 May 19;23(Suppl 4):381. doi: 10.1186/s12864-022-08602-9 (PMC9118565; doi:10.1186/s12864-022-08602-9)
Supplement: Supplementary file 1 — Additional file 1. [file 12864_2022_8602_MOESM1_ESM.docx]

**Table 1. Genomes used in this study.**

| **Species Name** | **Abbre.** | **Common**  **Name** | **Cultivar**  **/Ecotype** | **Assembly version** | **Source** |
| --- | --- | --- | --- | --- | --- |
| *Arabidopsis lyrata* | *A. lyrata* | - | - | v1.0 | Phytozome |
| *Arabidopsis thaliana* | *A. thaliana* | thale cress | Col-0 | TAIR 10 | TAIR |
| *Chlamydomonas reinhardtii* | *C. reinhardtii* | - | - | v5.5 | Phytozome |
| *Oryza sativa subsp. Japonica* | *O. sativa*  *(Japonica)* | rice | - | IRGSP-1.0 | EnsemblPlants |
| *Populus trichocarpa* | *P. trichocarpa* | poplar | - | v3.0 | Phytozome |
| *Selaginella moellendorfii* | *S. moellendorfii* | - | - | v1.0 | Phytozome |
| *Solanum lycopersicum* | *S. lycopersicum* | tomato | - | iTAGv2.4 | Phytozome |
| *Zea mays* | *Z. mays* | maize | B73 | 284 | Phytozome |

**Table 2. LncRNA distribution in this study.**

| **Species** | **lincRNA** | | **lncNAT** | | **All** |
| --- | --- | --- | --- | --- | --- |
|  | **New** | **Existed** | **New** | **Existed** |  |
| ***A. lyrata*** | 1,033 | 1,416 | 800 | 568 | 3,817 |
| ***A. thaliana*** | 354 | 502 | 4,710 | 1,189 | 6,755 |
| ***P. trichocarpa*** | 2,113 | 1,248 | 549 | 521 | 4,431 |
| ***S. lycopersicum*** | 1,484 | 2,000 | 1,154 | 641 | 5,279 |
| ***O. sativa*** | 3,429 | 2,422 | 715 | 332 | 6,898 |
| ***Z. mays*** | 2,286 | 3,683 | 883 | 1,469 | 8,321 |
| ***S. moellendorffii*** | 1,957 | 1,048 | 380 | 282 | 3,667 |
| ***C. reinhardtii*** | 182 | 193 | 284 | 118 | 777 |

**Table 3. SNP frequency (SNP count/1kb) in each species.**

| **Species** | **CDS** | **5’UTR** | **3’UTR** | **lincRNA** | **lncNAT** |
| --- | --- | --- | --- | --- | --- |
| *A.thaliana* | 0.3579 | 0.6401 | 1.6959 | 0.7834 | 1.3857 |
| *C.reinhardtii* | 0.0261 | 0.1248 | 0.1900 | 0.2248 | 0.1739 |
| *O.sativa* | 0.7513 | 1.3324 | 1.6767 | 1.6241 | 2.1296 |
| *P.trichocarpa* | 0.5201 | 2.1793 | 3.1666 | 3.6800 | 3.6754 |
| *S.moellendorfii* | 1.4488 | 4.7446 | 5.6944 | 8.1376 | 10.4330 |
| *S.lycopersicum* | 1.2043 | 2.9026 | 5.7262 | 4.3189 | 4.9987 |
| *Z.mays* | 1.9913 | 4.2512 | 4.6311 | 3.7658 | 4.5907 |

**Table 4. lncRNAs with TF binding site sequence originating from TE.**

| **TF*** | **Species** | **lncRNA amount** | **TE type*** |
| --- | --- | --- | --- |
| lySEP3 | *A. lyrata* | 178 | DNA/LINE/LTR/SINE/RC |
| LFYGR | *A. thaniana* | 1 | DNA |
| lfy | *A. thaniana* | 13 | DNA/LINE/LTR |
| LFYTERE | *A. thaniana* | 7 | DNA/LINE/SINE/LTR |
| NLP7_5 | *A. thaniana* | 1 | LTR |
| MADS29 | *O. sativa* | 51 | DNA/LINE/LTR |
| a-myc | *O. sativa* | 2 | LTR |
| P1 | *Z. mays* | 1 | LTR |

* TF, transcript factor

* TE type was from Repbase: <https://www.girinst.org/>
