# Supplementary figures and images for: Revealing the novel complexity of plant long non-coding RNA by strand-specific and whole transcriptome sequencing for evolutionarily representative plant species
Source: BMC Genomics. 2022 May 19;23(Suppl 4):381. doi: 10.1186/s12864-022-08602-9 (PMC9118565; doi:10.1186/s12864-022-08602-9)

**A**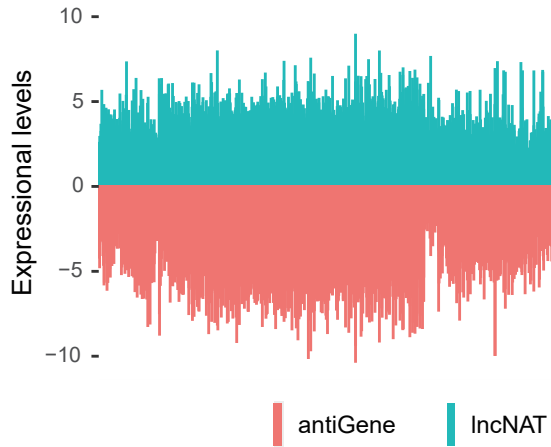**B**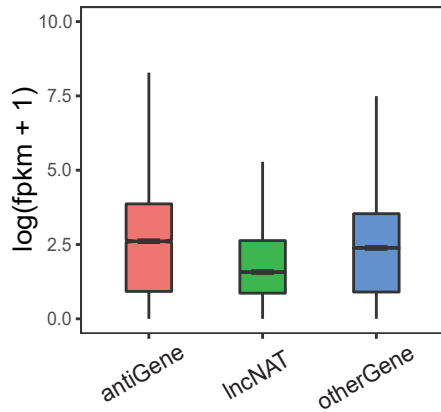

**A**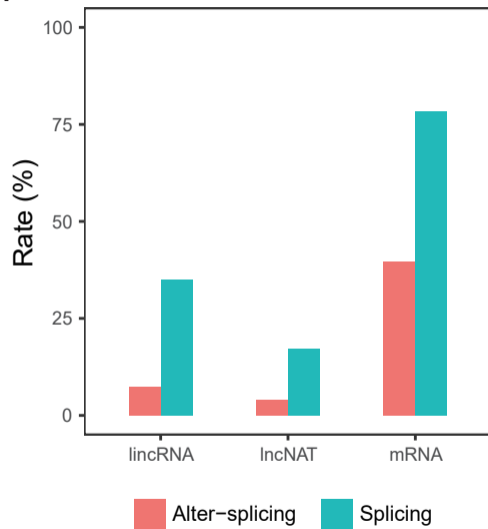**B**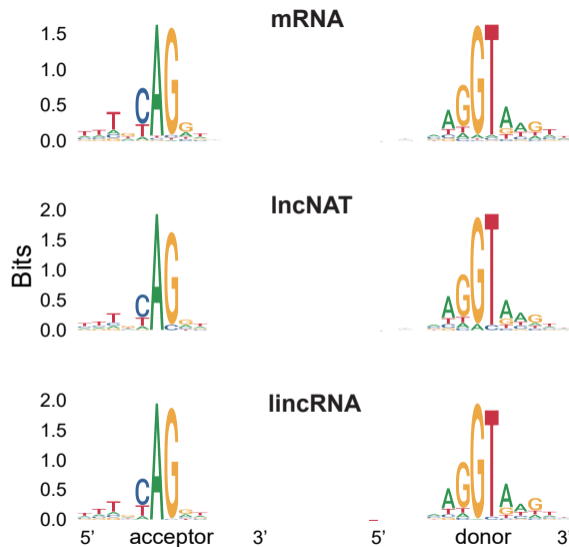

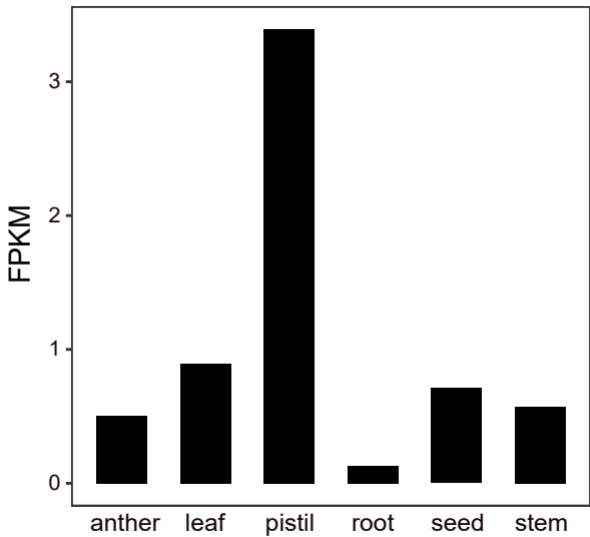

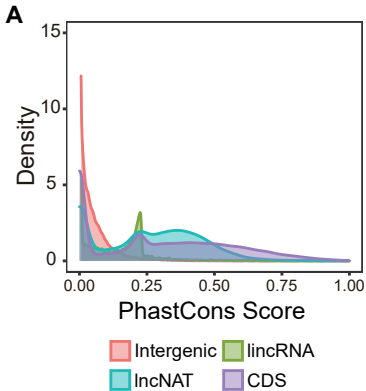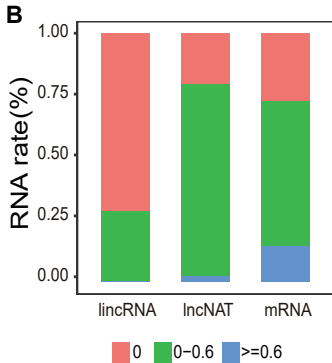

**A**

Expressional level

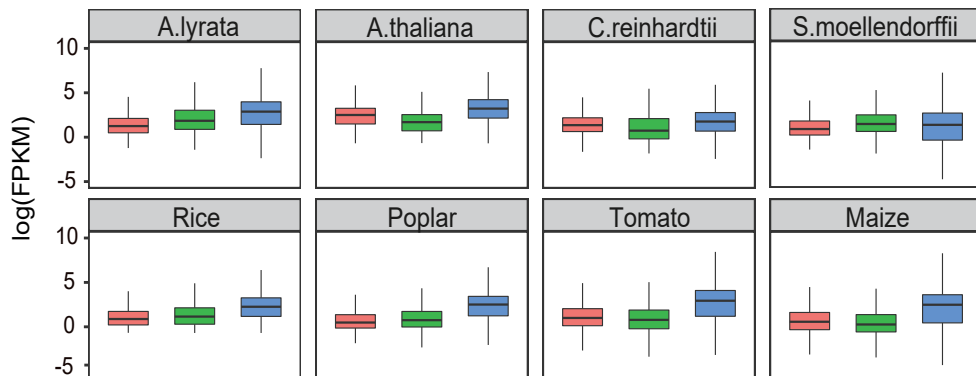**B**

Tissue specificity

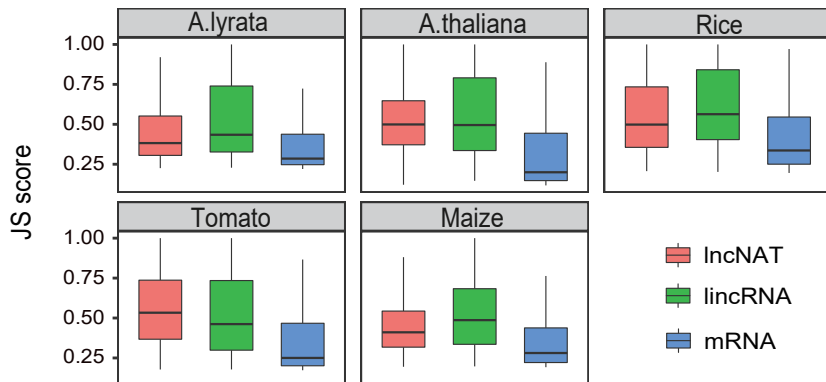

Supplement: Supplementary file 4 — Additional file 4: Figure S1. (A) Expressional levels of lncNATs and their antisense coding genes. The expressional levels were evaluated by log (FPKM+1). (B) Expressional levels of lncNAT, antisense coding genes, and coding genes without lncNATs expressing at their antisense strand. Figure S2. (A) Ratio of splicing and alternative splicing transcripts. (B) Base distribution of splicing acceptor and donor for lncRNAs and mRNAs. Figure S3. Expressional levels of lncRNA Osat_00007032 in rice. Figure S4. The distribution of PhastCons score in lncRNAs and coding genes. (A) The distribution of PhastCons score in lncRNAs, intergenic sequences and coding sequences of coding genes. (B) Percentage of lncRNAs and mRNAs with different PhastCons scores in total lncRNAs and mRNAs, respectively. Figure S5. Expressional patterns of lncRNAs and mRNAs in divergent species. (A) Expressional levels of lncRNAs and mRNAs in each species, and the numbers of y-axes were calculated by log10(FPKM). (B) Tissue-specificity of lncRNAs and mRNAs in each species with multiple tissues, and the y-axes stands for JS score. [file 12864_2022_8602_MOESM4_ESM.pdf]
